# Increased acetate production in *Synechocystis* sp. PCC 6803 strain engineered with an operon of phosphoketolase and phosphotransacetylase and further overexpression of acetate kinase

**DOI:** 10.1186/s12934-026-02964-5

**Published:** 2026-02-18

**Authors:** Stamatina Roussou, Peter Lindblad

**Affiliations:** Microbial Chemistry, Department of Chemistry-Ångström Laboratory, Uppsala University, Box 523, 75120 Uppsala, Sweden

**Keywords:** *Synechocystis* sp. PCC 6803, Acetate, Phosphoketolase, Phosphotransacetylase, Acetate kinase, High-density cultivation, Metabolic engineering

## Abstract

**Background:**

Photosynthetic microorganisms, such as cyanobacteria, are promising candidates for sustainable production of chemicals. Photosynthesis is a unique process where light energy is used to convert CO_2_ into carbon metabolites that sustain the cell`s metabolism. One of these products is acetate, a chemical with various applications in industry. Metabolic engineering can be used to increase the titer of extracellular acetate in the unicellular cyanobacterium *Synechocystis* sp. PCC 6803 (*Synechocystis*).

**Results:**

Simultaneous expression of phosphoketolase (PK) and phosphotransacetylase (Pta) resulted in an enhanced acetate titer in *Synechocystis* cells (Roussou et al. Metab Eng 88:250-260) [1]. In the present study these two enzymes were expressed in different locus in the genome as well as expressed in the same locus organized as a single operon. The latter design reached higher acetate production. Attempts to further optimize the production through the creation of fused protein did not result in significant higher values than 2.3 g/L previously reported. However, the production was further increased when acetate kinase (AckA) was additionally overexpressed. Cultivation of this strain in high density cultivation (CellDEG system) led to high levels of acetate with a maximum of 7.1 g/L cumulative acetate production after 12 days of experiment when the cultures were sampled every day.

**Conclusions:**

*Synechocystis* sp. PCC 6803 is a candidate for sustainable acetate production driven by sunlight and CO_2_. The high level of acetate production is result of combining genomic integration of heterogenous genes in the cell and overexpression of native genes through self-replication vector. The production level achieved through the high-density cultivation reveal the strain capabilities when the growth conditions are optimal.

**Supplementary Information:**

The online version contains supplementary material available at 10.1186/s12934-026-02964-5.

## Introduction

Cyanobacteria are photosynthetic microorganisms that are capable of using the sun light and CO_2_ to grow and multiply [2]. In times were the increased levels of CO_2_ in the atmosphere are contributing significantly to the climate crisis, the possibility to engineer and produce valuable chemicals through these specific bacteria has great potential on the road towards a more sustainable future. Several cyanobacteria have been engineered to produce a variety of chemicals including alcohols, terpenoids and hydrogen [3–6].

One of the most studied cyanobacteria is *Synechocystis* sp. PCC 6803, therefore *Synechocystis*, which can be genetically modified and facilitate bio-production of chemicals. Except the products that their biosynthesis has been added to its abilities, *Synechocystis* can also produce secondary metabolites as well as side products of the metabolism such as lactate, acetate and fumarate [7].

Acetate is a two-carbon molecule and the conjugative acid, acetic acid, which has several applications in the industry, as well as an intermediate product for the formation of other chemicals, for example, in the production of polyethylene terephthalate (PET). Acetic acid is also used as a food conservative (vinegar) [8]. Another application of acetate is as carbon source for growth, heterotrophic microorganisms such as *Escherichia coli* and *Pseudomonas* sp. thereby can transform this compound to useful bioproducts [9].

**Fig. 1:**
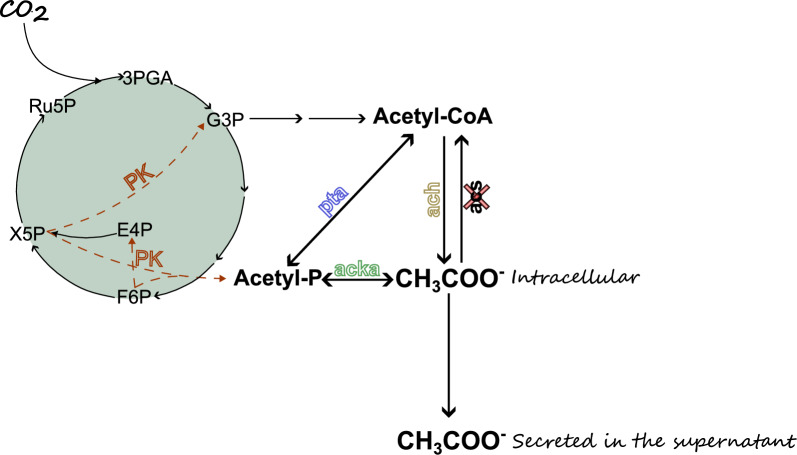
Simplified illustration of the Calvin-Benson-Bassham (CBB) cycle and acetate biosynthesis pathways. The native pathways are represented with black solid lines while the dashed orange lines represent the inserted phosphoketolase. The intermediates are shown through abbreviations 3PGA: 3-phosphoglycerate, G3P: glyceraldehyde-3-phosphate, DHAP: dihydroxyacetone phosphate, FBP: fructose-1,6-bisphosphate, SBP: sedoheptulose-1,7-bisphosphate, F6P: fructose-6-phosphate, S7P: sedoheptulose-7-phosphate, E4P: erythrose-4-phosphate, Xu5P: xylulose-5-phosphate, R5P: ribose-5-phosphate, Ru5P: ribulose-5-phosphate, RuBP: ribulose-1,5-bisphosphate. The following enzymes are also shown; pta, phosphotransacetylase; acka: acetate kinase, acs: acetyl-CoA synthetase, ach: acetyl-CoA hydrolase and PK: phosphoketolase. The red cross on the acs indicates the knock-out of the gene in all of the strains used in this study. Acetate is shown by its chemical form CH_3_COO^−^

The production of acetate can be either by petrochemical procedure or with the use of, mainly, heterotroph bacteria through for example gas fermentation or anaerobic oxidation of methane. Autotrophs, like *Synechocystis*, has also the natural capacity to produce acetate with only traces being transferred outside the cells. It is more common to monitor acetate production under dark anaerobic conditions [10]. In *Synechocystis*, acetate is mainly biosynthesized through Acetyl-P or Acetyl-CoA while there is also the possibility for acetate to be converted, irreversibly, to Acetyl-CoA through acetyl-coenzyme A synthetase (*acs*) with the parallel consumption of one ATP (Fig. 1). To form acetate using Acetyl-CoA as the starting biomolecule acetyl-CoA hydrolase (*ach*) is needed [11]. In parallel, the conversion of Acetyl-P to acetate is taking place by an acetate kinase (*acka*) and this is a reversible reaction contributing with the formation of ATP [12]. Finally, phosphotransacetylase (*pta*) is a necessary enzyme for the equilibrium between acetyl-P and acetyl-CoA since it is responsible for its reversible interconversion.

One option to increase the carbon flux to acetyl-P is through phopshoketolase and it has been reported that it can increase the extracellular pool of acetate in *Synechocystis* up to 40 times [1]. Phosphoketolase is converting xylulose 5-phosphate to acetyl-P and glyceraldehyde 3-phosphate and as well fructose-6-phosphate to acetyl-P and erythrose 4-phosphate [13]. There are strong indications for the existence of an endogenous phosphoketolase in cyanobacteria but, in general, it is expected to be active during darkness or heterotrophic and limiting nutrients conditions where it is found to regulate carbon fixation, tentatively through ATP-sensitivity. Even though a phosphoketolase has been characterized for *Synechococcus elongatus* PCC 7942, it is not the same case for *Synechocystis* [13, 14]. The phosphoketolase that resulted in increased acetate production under photosynthetic conditions is a heterologous enzyme originally from *Pseudomonas aeruginosa* (PKPa). There are more examples of increased production through an insertion and expression of a heterologous phosphoketolase [3, 10]. In the same study [1] it was established that further insertion of a phosphotransacetylase from *Bacillus subtilis* (BsPta) additionally doubled the acetate production in *Synechocystis*.

In the present study several approaches were used to optimize the expression of PKPa and BsPta; individual expression of the genes, expression under the same promoter, and production of fused proteins through different linkers. Previously, BsPta was expressed through a self-replication vector [1] whereas as in the present study the gene was inserted in the genome to achieve higher stability. A systematic approach was involved to end up to a stable, high acetate producing strain. Furthermore, the next steps of the acetate biosynthesis pathway were analyzed by overexpressing the native enzymes encoded by *acka* and *ach*. The two highest acetate producing strains were then cultivated in high density cultivation to investigate the maximal productivity of the strains under optimal growth conditions.

## Materials and methods

### Growth conditions and analysis for *Escherichia coli* and *Synechocystis* sp. PCC 6803

*Escherichia coli* (*E. coli*) cells were used for the subcloning. The medium used was LB and the cells were cultivated in liquid cultures or on agar plates containing LB and 1.5% agar (w/v). The temperature was always 37 °C. The cyanobacterium strain used in this study, *Synechocystis* sp. PCC 6803 (*Synechocystis*) was cultivated with BG11 medium [15] in liquid cultures or on agar plates (1.5% w/v). The temperature was 30 °C and the light intensity 65 μmol photons m^−2^ s^−1^. Two antibiotics (Sigma- Aldrich) were used for both of them, kanamycin and spectinomycin in a final concentration of 50 μg/ml. In the case where both of them were needed, half concentrations were used.

The growth of the cells was analyzed through optical density (OD) measured by a Varian Cary 50 BIO spectrophotometer. For the *E. coli* the OD was measured at 600 nm while for *Synechocystis* at 750 nm.

### Construction of plasmids and transformation to *Synechocystis* sp. PCC 6803

*Synechocystis* cells were transformed either with self-replicative plasmids or by genomic integration through integration vectors. The plasmids used for both transformation methods are shown in the Tables S2 and S3. The self-replicative plasmids were described previously [1] and transformed into *Synechocystis* by three-parental conjugation [16]. Colony PCR was followed to confirm the engineered strains.

In the case of genomic integration, the plasmids used were based on the pEERM vectors [17]. The homologous arms, that are necessary for the homologous recombination, were 1 kb upstream and downstream of the chosen locus. Overlap extension PCR was used to fuse the homologous arms with the expression and antibiotic cassettes. The PCR product was ligated through linear ligation with the backbone vector, containing the origin of replication expressed in *E. coli* cells. Colony PCR was used to verify correct clones. The protocol of natural transformation to *Synechocystis* is described in previously [1].

The primers used in this study are listed in the Table S.4. In case of amplification and overlap extension PCR the polymerase Phusion DNA polymerase (Thermo Fisher Scientific) was used while in colony PCR the DreamTaq DNA polymerase (Thermo Fisher Scientific) was chosen.

### Acetate production experiments in flasks

Seed cultures of the strains was inoculated in 6-well TC plates (Sarstedt) with BG11 with the appropriate antibiotic and grown in low light (30 μmol photons m^−2^ s^−1^) until mid-log phase. For the screening in short-term experiments an initial OD_750_ = 0.1 was used and the cells were cultivated in 20 ml BG11 supplemented with 50 mM NaHCO_3_ (Sigma-Aldrich) and containing the appropriate antibiotics in 100 ml Erlenmeyer flasks (VWR). The light intensity was 65 μmol photons m^−2^ s^−1^ and the temperature 30 °C. Every 2 days 2 ml cell cultures were removed from the culture and replaced with 2 ml of fresh BG11 supplemented with 500 mM NaHCO_3_. The OD and the acetate measurements were measured the same days. All experimental cultures were in biological triplicates.

For the long-term experiment in flasks, the initial OD was slightly higher (OD_750_ = 0.2) and furthermore on the sampling days the pH was adjusted with 37% HCl (Sigma-Aldrich) until pH values between 7 and 8.

### Acetate production in high density cultivation system

In order to cultivate selected strains in a high-density cultivation system, the HDC 6.10 starter kit (CellDEG, Germany) was used. The principle of this system is the creation of a saturated CO_2_ environment which in combination with an enriched medium (CD) and higher light intensity leads to higher and faster growth of the cells. The medium contained higher nutrient concentrations compared to BG11, both in essential nutrients and trace element and it was prepared according to the instruction of the manufacturer. A detailed protocol is available on www.protocols.io (10.17504/protocols.io.2bxgapn). For the creation of this environment, specialized 10 ml vials with porous hydrophobic membranes at the bottom and a reservoir equipped with the same membranes are used [18]. The reservoir was filled with 200 ml 3 M KHCO_3_/3 M K_2_CO_3_ in a 9:1 ratio according to manufacturer suggestions. The buffer was replaced every 3 days. The enriched medium was prepared with the addition of appropriate antibiotics [19]. The experiment was performed in triplicates and in order to achieve high light intensity a ‘Versatile Environmental Test Chamber’ (Sanyo) without humidifier was used, under 30 °C with the following settings: 250 μmol photons m^−2^ s^−1^ for the first day, increased to 490 μmol photons m^−2^ s^−1^ for day 2 and after day 3 the light intensity was 750 μmol photons m^−2^ s^−1^. The shaker used was the IKA KS 130 basic orbital shakers (ø ¼ 4 mm) and the CellDEG system was constantly shaken at 320 rpm.

The seed cultures were prepared as described above but after reaching the mid-log phase they were used to inoculate 20 ml BG11 buffered with 50 mM NaHCO_3_ and then cultivated under 65 μmol photons m^−2^ s^−1^ until OD_750_ = 1. The cells were pelleted through a 10 min centrifugation at 2700 xg and resuspended in fresh CD medium. The starting OD_750_ for the experiment was 0.2 and the final volume 10 ml. The growth was monitored every day through OD measurements. Two different sampling times were tested, the vials were sampled either every 24 h and 2 ml of the cultures were removed and replaced with the same volume of fresh medium or every 48 h. The acetate was quantified through the use of the sampled volume.

### Acetate quantification

For the acetate measurements 2 ml of supernatant was filtered with a 0.22 μm filter and further processed as detailed previously [1]. The total volume in the NMR tube was 0.6 ml containing 0.5 ml supernatant, 10 μl 50 μM HOOCC_6_H_4_COOK (internal standard) and filled up with D_2_O (both of them from Sigma Aldrich). When the acetate production was more than 2.5 g/l the volume was adjusted to 0.5 ml supernatant and 100 μl of internal standard. A JEOL (400YH magnet) Resonance 400 MHz spectrometer was used and the obtained ^1^H NMR spectra were analysed by Mestrenova software Version 6.0.2–5475 (Mestrelab).

## Results

### Expression of PKPa and BsPta as one operon increases acetate production compared to individual expression

**Fig. 2:**
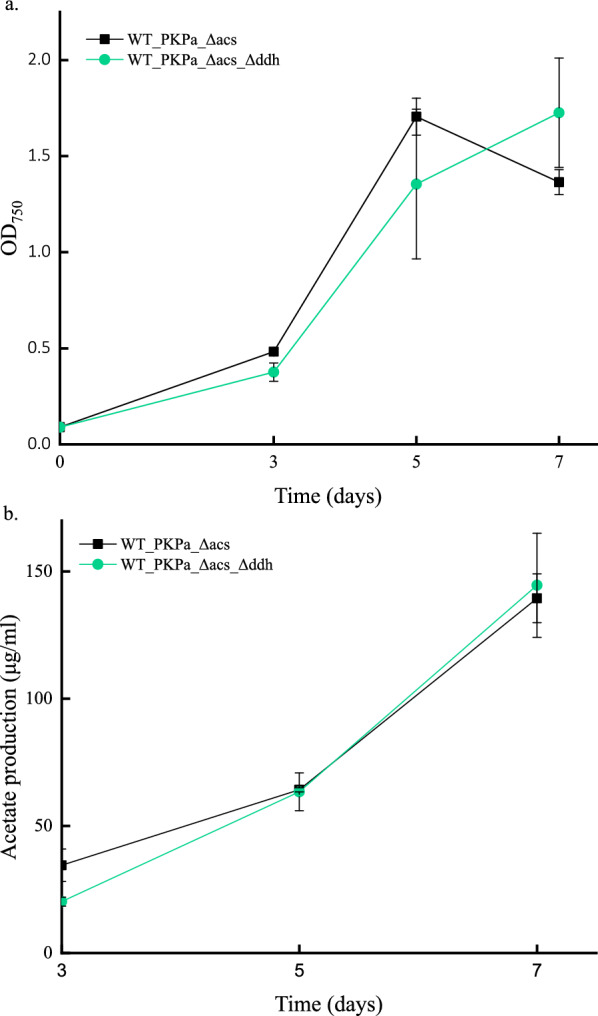
Acetate production in *Synechocystis* sp. PCC 6803 strains engineered with the *PKPa* gene (WT_PKPa_Δacs) compared to an extra knock out on the *ddh* locus (WT_PKPa_Δacs_Δddh). Shown are the growth of the cells (**a**) and the acetate production (**b**). The light intensity used to grow the cells was 65 μmol photons m^−2^ s^−1^ and on days 3, 5, and 7, 2 ml were sampled from the culture and replaced with 2 ml fresh BG11 with NaHCO_3_ (final concentration 50 mM) containing all the appropriate antibiotics. The 2 ml were used for OD and acetate measurements. The results are represented as mean ± SD of the three biological replicates

**Fig. 3:**
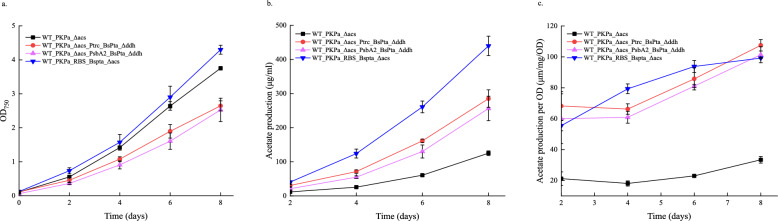
Acetate production in *Synechocystis* sp. PCC 6803 strains expressing PKPa (WT_PKPa_Δacs) compared to strains expressing additionally BsPta under two different promoters (WT_PKPa_Δacs_Δddh _Ptrc_BsPta and WT_PKPa_Δacs_Δddh _PsbA2_BsPta) and under the same operon (WT_PKPa _RBS_BsPta_Δacs). Shown are the growth of the cells (**a**), the acetate production (**b**), and the acetate production normalized by OD. The light intensity used to grow the cells was 65 μmol photons m^−2^ s^−1^ and every 2 days, 2 ml were sampled from the culture and replaced with 2 ml fresh BG11 with NaHCO_3_ (final concentration 50 mM) containing all the appropriate antibiotics. The 2 ml were used for OD and acetate measurements. The results are represented as mean ± SD of the three biological replicates

In previous study it was shown that acetate production in *Synechocystis* increases under photosynthetic conditions with a combined expression of a phosphoketolase (PK) and phosphotransacetylase (Pta) [1]. PK was inserted in the genome whereas Pta was overexpressed through a self-replication vector. However, genome integration offers more stable levels of expression. One common target for genomic integration in *Synechocystis* is the *ddh* locus, which encodes for D-lactate dehydrogenase, a side product of the cell´s metabolism. An integration vector targeting the ddh (*slr1556*) locus was constructed, carrying the spectinomycin antibiotic cassette, and transformed to the strain expressing the PK (WT_PKPa_Δacs). The newly constructed strain (WT_PKPa_Δacs_Δddh) showed no difference in the acetate production compared to the control strain (WT_PKPa_Δacs), neither statistically important differences in the growth of the cells during a short-term screening experiment (Fig. 1). With this clarified, the integration vector was further engineered to include the BsPta under the strong promoter P*trc* RiboJ or under the native promoter PsbA2. In parallel, an integration vector that includes the coexpression of PKPa and BsPta on *acs* locus under the same promoter (P*trc*RiboJ) was additionally constructed. The first two vectors were transformed to WT_PKPa_Δacs while the last one was transformed to WT *Synechocystis*. The growth and the acetate production of the obtained strains were measured (Fig. 2). It is noticeable that the strains overexpressing the Pta on the ddh locus WT_PKPa_Δacs_PtrcRiboJ_BsPta_Δddh and WT_PKPa_Δacs_PsbA2_ BsPta_Δddh grew a bit slower than the control strain expressing only the PK. The strain expressing both PK and BsPta on the same locus (WT_PKPa_RBS_BsPta_Δacs) grew similarly with the control strain. The acetate production followed a clear pattern where all the three strains expressing both PK and BsPta produced more than the control strain with specifically higher titer of production in the strain expressing both genes inserted in the same locus. This reduced the antibiotic need to only one antibiotic. When the acetate production was normalized by OD the same general pattern is noticeable with the exception that in the last day of the experiment (day 8) the acetate production levels between the three strains are not significantly different (Fig. 3).

### A fused construct between PKPa and BsPta did not affect the acetate production

**Fig. 4:**
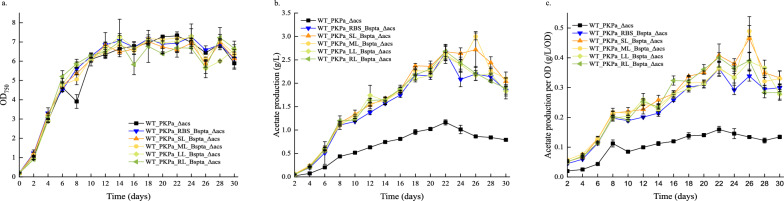
Acetate production in *Synechocystis* sp. PCC 6803 strains expressing PKPa and BsPta (WT_PKPa _RBS_BsPta_Δacs) in the same operon compared to strains containing a fused construct of the 2 genes joined by either different length of linkers (WT_PKPa _SL_BsPta_Δacs, WT_PKPa _ML_BsPta_Δacs, WT_PKPa _LL_BsPta_Δacs) or a rigid linker (WT_PKPa _RL_BsPta_Δacs). The light intensity used to grow the cells was 65 μmol photons m^−2^ s^−1^ and every 2 days, 2 ml were sampled from the culture and replaced with 2 ml fresh BG11 with NaHCO_3_ (final concentration 50 mM) containing all the appropriate antibiotics. At the same day the pH was adjusted with HCl to 7–8. The 2 ml were used for OD and acetate measurements. The results are represented as mean ± SD of the three biological replicates

To investigate the optimal co-expression of PK and Pta, an attempt to fuse them through linkers took place. Four new integration vectors were constructed where the RBS that facilitate the expression of Pta under the same promoter, was replaced with linkers. Three flexible linkers were tested; a short, a medium and a long linker (parts BBa_K24004-BBa_K24006). The short one was composed by the following 4 amino acids, Gly-Gly-Ser-Gly, the medium was the same sequenced duplicated and the long with the sequence triplicated. Lastly, an additional, rigid linker used previously [20] composed by the following 6 amino acids Leu-Gln-Ser-Arg-Leu-GLu was also included. The integration vectors containing the expression and antibiotic cassettes targeting the *acs* locus were transformed to WT *Synechocystis* cells. The growth of the cells was similar between the four strains expressing the fused constructs (WT_PKPa_SL_BsPta_Δacs, WT_PKPa_ML_BsPta_Δacs, WT_PKPa_LL_BsPta_Δacs and, WT_PKPa_RL_BsPta_Δacs), the WT_PKPa_RBS_BsPta_Δacs and the WT_PKPa_Δacs strain (Fig. 4). The acetate production and the acetate production normalized per OD between the strains were all very similar between the strain expressing both of the genes and higher than the strain expressing only the PK. In a total cultivation time of 30 days, an acetate production of 2.7 g/L, on day 26, was observed which is comparable to 2.3 g/L previously reported [1] indicating that the fused proteins that were constructed could not break this limit and additional engineer of the strains is necessary to investigate the potentially further increase of the acetate production.

### Overexpression of AckA increased the acetate production

**Fig. 5:**
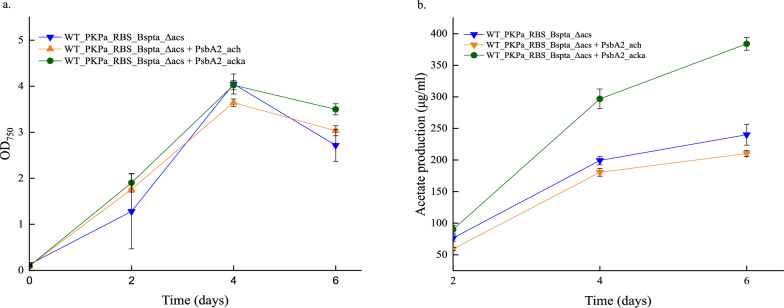
Acetate production in *Synechocystis* sp. PCC 6803 strains expressing PKPa and BsPta (WT_PKPa _RBS_BsPta_Δacs) compared to strains additionally expressing either the enzyme Ach or AckA. Shown are the growth of the cells (**a**), the acetate production (**b**), and the acetate production normalized by OD. The light intensity used to grow the cells was 65 μmol photons m^−2^ s-^1^ and every 2 days, 2 ml were sampled from the culture and replaced with 2 ml fresh BG11 with NaHCO_3_ (final concentration 50 mM) containing all the appropriate antibiotics. The 2 ml were used for OD and acetate measurements. The results are represented as mean ± SD of the three biological replicates

As shown in Fig. 1, phosphotransacetylase catalyzes the reversible conversion of acetyl-P to acetyl-CoA. It is already established that expression of PK and Pta increase the acetate pool and in order to investigate the effect(s) of overexpressing Acka or Ach, additional steps are required. In order to answer this question, the WT_PKPa_RBS_BsPta_Δacs was further engineered to overexpress Ach or AckA through self-replication vectors. These plasmids are listed in Table S.3 and they got transformed through conjugation to the selected strain. All three strains, WT_PKPa_RBS_BsPta_Δacs + PsbA2_ach, WT_PKPa_RBS_BsPta_Δacs + PsbA2_acka, and WT_PKPa_RBS_BsPta_Δacs grew comparably as shown in Fig. 5. However, different production levels of acetate were noticed. The strain overexpressing the AckA showed significantly higher production compared to the other two. Interestingly, the strain that overexpress Ach, performed slightly worse than the control strain, WT_PKPa_RBS_BsPta_Δacs. These results showed that it is possible to increase further the acetate production by overexpression of AckA.

### High-density cultivation enhanced acetate production up to 7.1 g/L in *Synechocystis* sp. PCC 6803

**Fig. 6:**
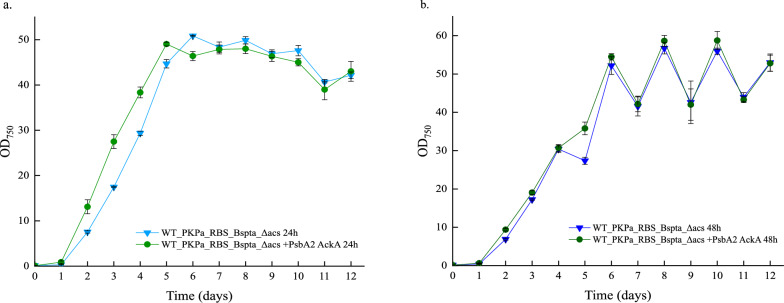
Growth of *Synechocystis* sp. PCC 6803 strains engineered to express PKPa and BsPta as a single operon and strain further engineered to overexpress AckA during high-density cultivation for 12 days. OD measurements were performed every day and the cultures were sampled either once per day (**a**) or every 2 days (**b**). The sampling was 20% of the final volume and the same volume of fresh CellDEG media with the appropriate antibiotics was added in the vials. Every 3 days the 3 M carbonate buffer was replaced. The results are represented as mean ± SD of the three biological replicates

The maximal production of acetate in flasks observed is 2.7 g/L, after 26 days of cultivation. However, the OD_750_ of the cell culture did not reach higher than 7. It may be interesting to investigate acetate levels in a high-density cultivation system, where a saturated CO_2_ atmosphere, higher light intensity and nutrient-enriched medium will promote cell growth and potentially acetate production. This may be achieved using the CellDEG cultivation system fulfilling all above criteria. The highest performing strain was chosen, the strain expressing PK and Pta as a single operon and additionally overexpress AckA, WT_PKPa_RBS_BsPta_Δacs + PsbA2_acka, with WT_PKPa_RBS_BsPta_Δacs as control strain, and grown in high-density conditions. The strains were cultivated in 2 triplicate each. The difference between these two triplicates is that one was sampled every day (24 h) and the other every second day (48 h). The aim of this process was to examine the effects of a more continuous dilutions of the high-density cultures in both growth and acetate production, since 20% of cell culture was sampled and replaced with fresh medium including antibiotics. The effects on the growth of the cells are very clear in both sampling methods as shown in Fig. 6. In the case where the cells were sampled every day, the OD_750_ reached up to 50 in day 6, the growth between days 1 and 5 being linear and then the cells entered a stationary phase before the OD_750_ started slowly dropping (days 10 to 12). Both strains showed comparable growth but it is important to notice that WT_PKPa_RBS_BsPta_Δacs + PsbA2_acka stopped growing earlier than the control strain. The OD_750_ start gradually decreasing after day 5 and remained lower than for the WT_PKPa_RBS_BsPta_Δacs strain for the remaining time of the experiment. The dilution effects due to sampling is more clear in the 48 h sampling pattern. In this case the OD_750_ reached higher values, reaching almost 60 (58.6) on day 8. The cells grew in a linear way between days 1 to 4, while after this time point the dilution effect is clear, since the OD_750_ significantly dropped the day after the sampling and increased again. The cells grew very similar with the strain overexpressing additionally the AckA consistently showing slightly higher OD_750_ values compared to the control strain. This way the cells grew to higher OD_750_ compared to being sampled once per day. In both cases, a small log phase is noted between day 0 and day 1.

**Fig. 7:**
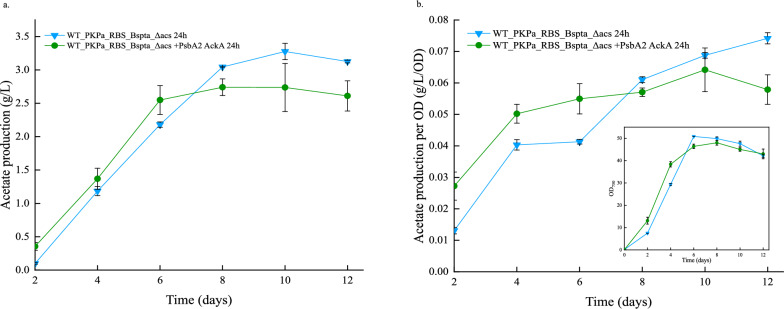
Acetate production in *Synechocystis* sp. PCC 6803 strains engineered to express PKPa and BsPta as a single operon and strain further engineered to overexpress AckA during high-density cultivation for 12 days. Shown are the production (**a**) and the production normalized per OD, in the graph is also included the growth (**b**). Shown are the results for every 2nd day while the triplicates were sampled every day. The sampling was 20% of the total volume and the same volume of fresh CellDEG media with the appropriate antibiotics was added in the vials. Every 3 days the 3 M carbonate buffer was replaced. The results are represented as mean ± SD of the three biological replicates

**Fig. 8:**
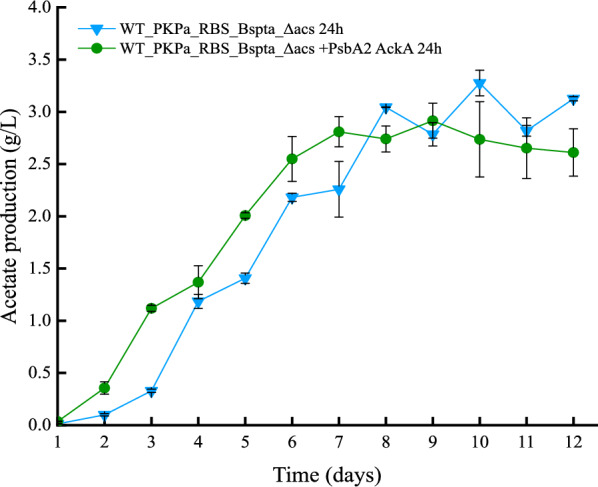
Acetate production in *Synechocystis* sp. PCC 6803 strains engineered to express PKPa and BsPta as a single operon and strain further engineered to overexpress AckA during high-density cultivation for 12 days. The cultures were sampled once per day. The sampling was 20% of the final volume and the same volume of fresh CellDEG media with the appropriate antibiotics was added in the vials. Every 3 days the 3 M carbonate buffer was replaced. The results are represented as mean ± SD of the three biological replicates

**Fig. 9:**
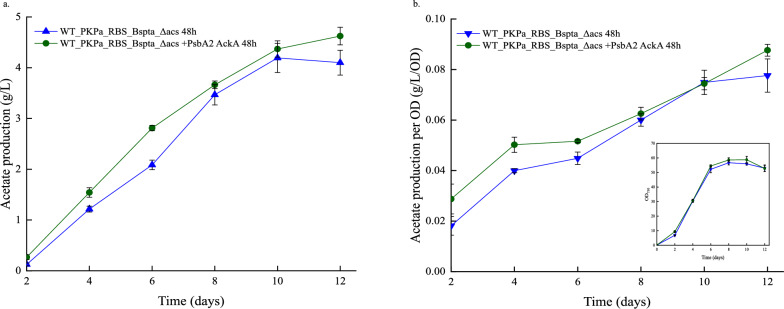
Acetate production in *Synechocystis* sp. PCC 6803 strains engineered to express PKPa and BsPta as a single operon and strain further engineered to overexpress acka during high-density cultivation for 12 days. Shown are the production (**a**) and the production normalized per OD in the graph is also included the growth (**b**) every second day. The sampling every 2 days, was 20% of the total volume and the same volume of fresh CellDEG media with the appropriate antibiotics was added in the vials. Every 3 days the 3 M carbonate buffer was replaced. The results are represented as mean ± SD of the three biological replicates

The acetate production reached high values in both triplicates. More specifically, in the experiment where the cell cultures were sampled every day, 3.2 g/L at the 10th day of cultivation, in the control strain were measured (Fig. 7). The acetate production is higher in that strain compared to the strain overexpressing additionally the AckA after day 8 (Fig. 7 and Fig. 8). This correlates with the decrease in the OD_750_ as discussed above. The same pattern is also observed in the production normalized per OD (Fig. 7). However, this is not noticed in the cultures sampled every 48 h (Fig. 9). The strain WT_PKPa_RBS_BsPta_Δacs + PsbA2_acka showed a higher production through all the cultivation days reaching 4.6 g/L on the last day of the experiment while the control strain reached 4.2 g/L on day 10. The acetate production normalized per cell followed the same pattern.

**Fig. 10:**
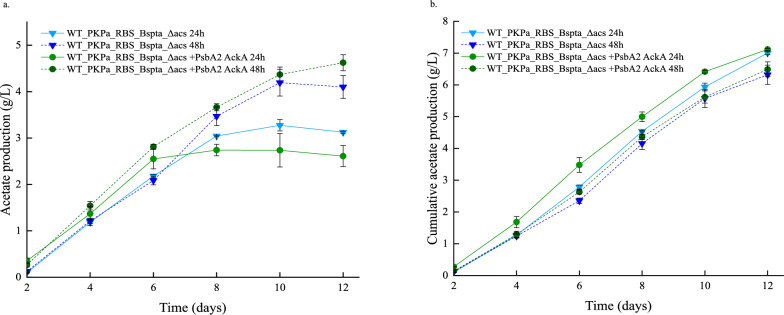
Acetate production *Synechocystis* sp. PCC 6803 strains engineered to express PKPa and BsPta as a single operon and strain further engineered to overexpress AckA during high-density cultivation for 12 days. Shown are the acetate production in vial (**a**) and the cumulative production (**b**) for both the triplicates sampled every day and every second day. The results are represented as mean ± SD of the three biological replicates

The comparison between the in-flask acetate production results showed that overall, the titers are higher when the sampling takes place every 2 days for both strains (Fig. 10). However, this pattern changes when the cumulative acetate production is calculated. The cumulative production includes the in-flask acetate plus the acetate that was removed from the flask at each sampling time point. In this case it is noticeable that when the cultures are sampled more often (once per day compared to once every second day) the production is higher. The strain WT_PKPa_RBS_BsPta_Δacs + PsbA2_acka reached 7.1 g/L cumulative production in the last day of cultivation, followed by the control strain that reached 7 g/L on the same day. During the previous day of cultivation, the cumulative production of the WT_PKPa_RBS_BsPta_Δacs + PsbA2_acka strain is higher than the control and this result could be due to lower OD_750_ and lower acetate production in the last days of the cultivation as well. The same strain showed also the highest cumulative production when sampled every 2 days, 6.5 g/L while the control strain reached 6.3 g/L in the end of the cultivation period.

## Discussion

In the present work we explored different pathways to increase the acetate production in *Synechocystis* and we managed to reach 7.1 g/L cumulative acetate production over 12 days of high-density cultivation, a significant high level of production, especially for photosynthetic microorganisms [2]. Acetate biosynthesis in *Synechocystis* is mainly based in acetyl-P and acetyl-CoA. In this study acetyl-P was enhanced by phosphoketolase (PK) and the acetate pathway was further enhanced through the overexpression of phosphotransacetylase (Pta) and acetate kinase (AckA), both key enzymes for the process.

In earlier study it was established that PK and Pta increased the acetate production up to 2.3 g/L through 12 days of cultivation [1]. This was possible since PK is a glycolysis-related enzyme that drags carbon from the main fixation pathway, the CBB cycle, to acetyl-P with simultaneously production of either G3P or E4P. This is depending on the substrate of the enzyme, that can be either X5P or F6P, respectively [21]. The specific PK used in the study (PKPa) was a codon optimized gene originally from *P. aeruginosa* [1]. Metabolomics analysis showed accumulation of acetyl-P in the cells expressing the PKPa and stability over the levels of acetyl-CoA. These results may explain why Pta led to higher yields of acetate. The pta used in this study is a codon optimized version originally from *B. subtilis* (BsPta).

In order to address plasmid instability and variation in production levels, a decision to insert BsPta in the genome was made. One candidate genome locus was *ddh*, coding for D-lactate dehydrogenase that synthesize lactate, a side product of the cell´s metabolism. The locus was knocked out resulting to a new strain, WT_PKPa_Δacs_ Δddh, which produced the same acetate levels as the control strain (WT_PKPa_Δacs). This result indicated that acetate and lactate synthesis do not compete with each other. With this background knowledge, *BsPta* was inserted in the *ddh* locus under the control of either P*sbA2* or PtrcRiboJ promoter in the control strain (WT_PKPa_Δacs). At the same time, another construct was developed where PK and Pta were expressed through a single operon at the *acs* locus, under the control of P*trc*RiboJ and successfully inserted in *Synechocystis* WT. This reduced the need of antibiotics by half.

The three strains that occurred after these transformations were compared to WT_PKPa_Δacs, in terms of growth and acetate production. It is important to notice that both strains where BsPta was expressed individually in the *ddh* locus grew slower (WT_PKPa_Δacs_Ptrc_BsPta_ Δddh and WT_PKPa_Δacs_PsbA2_BsPta_ Δddh) while the other strain (WT_PKPa_RBS_BsPta_Δacs) grew slightly better. All three stains expressing both PK and Pta produced more acetate than the control strain, while the strain that expressed both of the genes in the same locus produced more than the other two. These two strains showed comparable results both in acetate production and on acetate production normalized by OD. Even though strain WT_PKPa_RBS_BsPta_Δacs showed significantly higher acetate production per volume, only on days 4 and 6 was a similar effect observed when expressed per OD. Different hypothesis may be proposed to explain these results. The two different expression units’ approach could have led to metabolic burden to the cells and occupation of the translation machinery. The one mRNA that was produced from the strain WT_PKPa_RBS_BsPta_Δacs could potentially led to higher translation efficiency. Finally, it could be that the levels of the enzyme developed better stoichiometry through this approach and that leads to higher production and slightly better growth. One theory for the last result may be that the less accumulation of acetyl-P led to better growth as there are indications of the role of acetyl-P in signaling and as a global regulator [22, 23]. Another reason explaining the higher acetate production in the single operon approach could be a tentative co-localization of the expressed proteins. Based on this note, 4 different strains were developed with the aim to study the acetate biosynthesis of a fused protein. For this purpose, 3 flexible linkers were chosen from the BioBrick library, a short (4 amino acids), a medium (8 amino acids) and a long linker (12 amino acids) as well as a rigid linker (6 amino acid) earlier used in the lab [20]. Two control strains were used for this study, WT_PKPa_Δacs and WT_PKPa_RBS_BsPta_Δacs and the, six in total, strains were cultivated for 30 days. To be possible to maintain the cultivation for this time period, the pH was controlled with HCl and adjusted between the values 7–8. This is necessary since every 2 days 2 ml of culture were removed and replaced with fresh medium containing NaHCO_3_ in a final concentration 50 mM and this leads to an alkaline pH in the culture [24]. During this long-term cultivation of the cells, similar growth pattern was noticed. The acetate production and the acetate production normalized by OD followed the same pattern. The strain expressing only PKPa produced less than the other five strains with no significant differences between them. This showed that the fused proteins did not perform better than the single operon in this specific design. Several explanations can be proposed over this result. Both of the enzymes are large, PKPa is 89.4 kDa and BsPta is 35 kDa, and the linkers chosen were relatively short in length. It has been shown that longer linkers are more hydrophilic and the length influence significantly the protein activity [25]. So, it may be that the new protein was not as flexible or as stable as desired. If the terminals of the individual proteins showed restrains it could also be that the fused protein is unstable and therefore breaks into the initial proteins, leading to eventually same levels of acetate synthesis. Another result that is safe to be concluded is that substate diffusion is already fast and the bottleneck of acetate production is not in the protein expression.

Following this approach, the last two enzymes of the acetate pathway were overexpressed on self-replicated vectors. The background strain for this study was WT_PKPa_RBS_BsPta_Δacs. These two enzymes, Ach and AckA, showed different effects on acetate titers. Overexpression of Ach slightly decreased the acetate production compared to the control strain while AckA showed significant increase. Ach is an enzyme that converts acetyl-CoA to acetate. When PKPa is active in *Synechocystis* it is shown that acetyl-CoA levels did not increase compared to in the WT cells [1]. However, it is not shown, to our knowledge, how additional expression of BsPta influenced the acetyl-CoA pool, but if the same pattern is active, then it could be that there is no acetyl-CoA accumulation available to increase the levels of acetate. Acetyl-CoA is one of the major metabolites of the cell so it could be a regulatory mechanism controlling the amount of it available for acetate biosynthesis since it is a side product for the metabolism. AckA catalyzes the reaction acetyl-P + ADP ↔ acetate + ATP, so, in collaboration with BsPta controls the levels of acetyl-P. During darkness and anaerobic conditions, AckA is responsible for the biosynthesis of acetate [10] while it is also generating ATP. The increase of acetate level in this strain, shows that the route Pta + AckA is more flavored than the Pta + Ach, in a condition where PKPa and BsPta are also present. The same route (Pta and AckA) is controlling the acetate fluxes in other microorganisms as well, such as *E. coli* [26] and these two genes are organized in the same operon [27], a condition not present in *Synechocystis*, since *pta* and *ach* are encounter in the same locus. However, in this study PK and Pta are heterogenous enzymes and this leads to different results compared to the native regulated system. These results are also emphasizing the importance of BsPta for acetate biosynthesis, since when *acka* and *ach* were overexpressed in WT_PKPa_Δacs strain, the production did not increase [1].

The new, higher producing strain, WT_PKPa_RBS_BsPta_Δacs + PsbA2_acka, and the previous higher strain, WT_PKPa_RBS_BsPta_Δacs, were used for small scale high-density cultivation. For this purpose, the CellDEG cultivation system was used, in order to achieve optimal conditions in terms of CO_2_ and light distribution [18]. Furthermore, two sampling systems were tested, where the cultures were sampled every day (24 h) or every second day (48 h). It has been shown that frequent removal of the product, leads to higher productivity, at least in case of butanol production [28] and it will be interesting to investigate if this was also the case for a native product of the metabolism such as acetate. In both cases, the cells reached high OD (Fig. 6) but it was higher in the case of sampling the culture every 48 h, since it reached almost OD_750_ = 60. The sampling volume was 20% of the culture and the same volume was replaced with fresh CD medium with the appropriate antibiotics. In the same case it is noticeable the dilution effect in the OD, especially after day 6 while when the cells were sampled every 48 h, seems to adjust better to the dilution process. It is also noticed that in the 24 h sampling condition, WT_PKPa_RBS_BsPta_Δacs + PsbA2_acka start growing slower compared to the control strain after day 6, while they grew similarly in the 48 h sampling. This effect is also shown on acetate production, as WT_PKPa_RBS_BsPta_Δacs + PsbA2_acka start to produce less than the control after day 8, both per titer and normalized per OD, while this is not the case in 48 h sampling, where the strain produced more than the control throughout the experiment.

It is clear that the strain WT_PKPa_RBS_BsPta_Δacs + PsbA2_acka seems to be affected more than the strain WT_PKPa_RBS_BsPta_Δacs when the cells are diluted 20% every day. In the 24 h sampling it seems that the first strain start growing slower after day 4 already, while the control strain after day 6. This rapidly decrease in the growth rate may explain the lower acetate yield, in combination of the continuous dilution. It is not clear why the overexpression of *acka* led faster to this result but one theory may be that the ATP balance in the cells is disrupted, since AckA synthesize acetate with the simultaneous production of ATP. Also, it is shown that in *Synechococcus elongatus* PCC 7942, PK activity is inhibited by ATP, so the effect of a negative feedback regulation cannot be excluded [14].

The acetate production reached the highest record, to our knowledge, in photosynthetic conditions, 4.6 g/L on day 12, when using strain WT_PKPa_RBS_BsPta_Δacs + PsbA2_acka sampled every 2 days. The control strain reached 4.1 g/L, while a bit lower was the production in the 24 h sampling (2.6 g/L and 3.1 g/L, respectively). The same strain reached 1.4 g/L acetate production at the same time period when cultivated in flasks, the CellDEG cultivation system increased the production almost 3 times due to a combination of increased light intensity and supply of carbon and nutrients. When the cumulative production was calculated, the opposite is noticed, where the cultures sampled every day showed eventually higher cumulative acetate production. Despite the drop in the acetate synthesis, WT_PKPa_RBS_BsPta_Δacs + PsbA2_acka showed the highest cumulative production, up to 7.1 g/L, followed by the control strain (7 g/L). The same pattern is followed when the cultures were sampled every 2 days reaching 6.5 g/L and 6.3 g/L, respectively. These results are among the highest production levels recorded in cyanobacteria. When it comes to *Synechocystis* especially, the highest titer we could find in the literature is citramate which reached 6.35 g/L in bioreactor cultivation [29] after 100 h (4.2 days). These results show the potential of cyanobacteria as cell factories and in parallel the significant role of high-density cultivation systems where high titers and production rates can be recorded [18, 19]. Even though, the acetate titers reported here are lower than the heterotroph levels, e.g., 29.4 g/L produced by microbial electrosynthesis [30], the potential of *Synechocystis* as acetate producer is clear. This can be further enhanced by a combination of systematic engineering approaches and efficient growth systems with effective acetate removal.

## Conclusions

The present work focused on different approaches to optimize acetate production from *Synechocystis* and explore the acetate biosynthesis in high-density cultivation. Acetate is a side product of the metabolism and necessary precursors are acetyl-P and acetyl-CoA. The production is highly increased though expression of a heterogenous phosphoketolase, which drags more carbon from the CBB circle to acetyl-P, and a heterogenous phosphotransacetylase, responsible for the equilibrium of acetyl-P and acetyl-CoA. However, overexpression of both enzymes under the same promoter and at the same genetic locus did not increase the production further. This bottleneck was solved by the overexpression of the native acetate kinase. The two higher producing strains were cultivated in the CellDEG system, which allows high-density cultivation. The cumulative acetate production reached 7.1 g/L when the cultures were sampled every day, showing the great potential of *Synechocystis* as a cell factory when the growth conditions are optimal.

## Supplementary Information

Supplementary Material 1.

## Data Availability

The datasets used during the current study are available from the corresponding author on reasonable request.
