# Supplementary material for: Increased acetate production in Synechocystis sp. PCC 6803 strain engineered with an operon of phosphoketolase and phosphotransacetylase and further overexpression of acetate kinase
Source: Microb Cell Fact. 2026 Feb 18;25:61. doi: 10.1186/s12934-026-02964-5 (PMC12930666; doi:10.1186/s12934-026-02964-5)
Supplement: Supplementary file 1 — Supplementary Material 1. [file 12934_2026_2964_MOESM1_ESM.pdf]

Increased acetate production in *Synechocystis* sp. PCC 6803 strain engineered with an operon of phosphoketolase and phosphotransacetylase and further overexpression of acetate kinase

Stamatina Roussou and Peter Lindblad*

stamatina.roussou@kemi.uu.se

peter.lindblad@kemi.uu.se

Microbial Chemistry, Department of Chemistry-Ångström Laboratory, Uppsala University, Box 523, SE-75120 Uppsala, Sweden

Additional File 1

Tables S.1-S.4

References

Table S.1: Genetic engineered *Synechocystis* sp. PCC 6803 strains used in this study

Synechocystis strain	Relevant Genotype	References
WT_PKPa_Δacs	Δacs:: (P*trc*RiboJ-***PKPa***-T)Km^R	Roussou *et al.*, 2025
WT_PKPa_Δacs_Δddh	Δacs:: (P*trc*RiboJ-***PKPa***-T)Km^R, Δddh::Sm^R	This study
WT_PKPa_Δacs_Δddh_Ptrc_BsPta	Δacs:: (P*trc*RiboJ-***PKPa***-T)Km^R, Δddh:: (PtrcRiboJ-***BsPta***-T)Sm^R	This study
WT_PKPa_Δacs_Δddh_PsbA2_BsPta	Δacs:: (P*trc*RiboJ-***PKPa***-T)Km^R, Δddh::(PsbA2-***BsPta***-T)Sm^R	This study
WT_PKPa_RBS_Bspta_Δacs	Δacs:: (P*trc*RiboJ-***PKPa***-RBS-***BsPta***-T)Km^R	This study
WT_PKPa_SL_Bspta_Δacs	Δacs:: (P*trc*RiboJ-***PKPa***-SL*-***BsPta***-T)Km^R	This study
WT_PKPa_ML_Bspta_Δacs	Δacs:: (P*trc*RiboJ-***PKPa***-ML*[1]-***BsPta***-T)Km^R	This study
WT_PKPa_LL_Bspta_Δacs	Δacs:: (P*trc*RiboJ-***PKPa***-LL*[2]-***BsPta***-T)Km^R	This study
WT_PKPa_RL_Bspta_Δacs	Δacs:: (P*trc*RiboJ-***PKPa***-RL*[3]-***BsPta***-T)Km^R	This study

*Part: BBa_K243004 Short Linker (Gly-Gly-Ser-Gly)
*[1]Part: BBa_K243005 Middle Linker (Gly-Gly-Ser-Gly) x2
*[2]Part: BBa_K243006 Long Linker (Gly-Gly-Ser-Gly) x3
*[3]Rigid linker (Leu-Gln-Ser-Arg-Leu-Glu), (Oliveira and Lindblad, 2011)
Antibiotic markers: Km = kanamycin, Sm = spectinomycin
The strong Terminator BBa_B0015 is indicated by T in the expression unit
With bold are indicated the expressed genes

Table S.2: Plasmids targeting *Synechocystis* sp. PCC 6803 genome used in this study

Backbone Vector	Integration site	Antibiotic resistance/genetic construct	Reference
IVE Δacs-PKPa-RBS-BsPta	*acs* (*sll0542*)	Km/ P*trc*RiboJ-***PKPa***-RBS-***BsPta***-T	This study
IVE Δacs-PKPa-SL-BsPta	*acs* (*sll0542*)	Km/ P*trc*RiboJ-***PKPa***-SL*-***BsPta***-T	This study
IVE Δacs-PKPa-ML-BsPta	*acs* (*sll0542*)	Km/ P*trc*RiboJ-***PKPa***-ML*[1]-***BsPta***-T	This study
IVE Δacs-PKPa-LL-BsPta	*acs* (*sll0542*)	Km/ P*trc*RiboJ-***PKPa***-LL*[2]-***BsPta***-T	This study
IVE Δddh	*ddh* (*slr1556*)	Sm	This study
IVE Δddh-Ptrc_BsPta	ddh (slr1556)	Sm/ PtrcRiboJ-***BsPta***-T	This study
IVE Δddh-PsbA2_BsPta	ddh (slr1556)	Sm/ PsbA2-***BsPta***-T)	This study

*Part: BBa_K243004 Short Linker (Gly-Gly-Ser-Gly)
*[1]Part: BBa_K243005 Middle Linker (Gly-Gly-Ser-Gly) x2
*[2]Part: BBa_K243006 Long Linker (Gly-Gly-Ser-Gly) x3
*[3]Rigid linker (Leu-Gln-Ser-Arg-Leu-Glu), (Oliveira and Lindblad, 2011)
Antibiotic markers: Km = kanamycin, Sm = spectinomycin
The strong Terminator BBa_B0015 is indicated by T in the expression unit
With bold are indicated the expressed genes

Table S.3: Plasmids and *Synechocystis* sp. PCC 6803 strains used in this study

Plasmid	Promoters and enzymes	*Synechocystis* strains	References
PsbA2+ach	P*psbA2*, acetyl-CoA hydrolase (*ach*)	WT_**PKPa**_RBS_**BsPta**_Δacs +PsbA2_**ach**	Roussou *et al.*, 2025/This study
PsbA2+acka	P*psbA2*, acetate kinase (*acka*)	WT_**PKPa**_RBS_**BsPta**_Δacs +PsbA2_**acka**	Roussou *et al.*, 2025/This study

With bold are indicated the expressed genes

Table S.4: Primers used in this study

Name	Sequence	Reference
US_acs_Pho_F	/5Pho/TTTCCACTTCACTTGGTT	Roussou **et al.**, 2025
US_acs_Ptrc_R	GAGCGCTCACAATTGTCAACAGCTCGGTTCTCCGTC AAAGTCT	Roussou **et al.**, 2025
DS_acs_R	CAAATTAGCCAAACCCAC	Roussou **et al.**, 2025
DSsll0542_linker_F	GTTAATTGGTTGTAACACTGGCAGAGC	This study
US_ddh_F	/5Pho/AAACCACTGGGCCAGTAG	Roussou **et al.**, 2025
US_ddh_Ptrc_R	GAGCGCTCACAATTGTCAACAGCTCGACGATTATGG GAAGTAGTT	Roussou **et al.**, 2025
DS_ddh_Sm_F	TGGTGAGAATCCAAGCCTCGAGCTGGGTTAGAAAAT ATCAATGTTAACT	Roussou **et al.**, 2025
DS_ddh_R_	CGGACTATTTGGTAGAACA	Roussou **et al.**, 2025
Ptrc_F	/5Phos/GAGCTGTTGACAATTGTGAGCG	Roussou **et al.**, 2025
Ptrc_acs_F	CCATTAAAGACTTTGACGGAGAACCGAGCTGTTGAC AATTGTG	Roussou **et al.**, 2025
Ptrc_RiboJ_R	CATTTTTTTCCTCCTTCTAGT	Roussou **et al.**, 2025
Ptrc_ddh_F	TTCTAAACTACTTCCCATAATCGTCGAGCTGTTGAC AATTGTG	Roussou **et al.**, 2025
PsbA2_ddh_F	AACTACTTCCCATAATCGTCTCCGCCAGGTAAACTC TTC	This study
KmR_F	GGTTGCATTCGATTCCTGTT	Roussou **et al.**, 2025
Km_Pho_R	/5Pho/CAATTCTGATTAGAAAAACTCATCGAGC	This study
SmR_ddh_R	TAGTTAACATTGATATTTTCTAACCCAGCTCGAGGC TTGGATT	Roussou **et al.**, 2025
SmR_R	/5Pho/CAGCTCGAGGCTTGGATT	Roussou **et al.**, 2025
PK_PtrcRiboJ_F	TAGAAGGAGGAAAAAAATGATGTGGAGTCATCCTC AG	Roussou **et al.**, 2025
PK_nsc_Pho_R	/5Pho/ACGCAATAAGGCGGGATCGCC	This study
pkpa_mid_R	CGGTATGGGGATTCACATG	Roussou **et al.**, 2025
pkpa_seq_R	CAGGCGGACACCACGGCTTCT	Roussou **et al.**, 2025
Pta_colony_R	ATGGCCACGCGGGGTTCA	Roussou **et al.**, 2025
SL_BsPta_F	GGTGGTAGTGGTGCCGACTTGTTTAGCACTGT	This study
ML_BsPta_F	GGTGGGAGTGGGGGAGGAAGCGGTGCCGACTTGTT TAGCACTGT	This study
LL_BsPta_F	GGTGGTAGTGGTGGTGGGAGTGGGGGAGGA	This study
RL_BsPta_F	CTGCAAAGCCGCCTAGAAGCCGACTTGTTTAGCACT GT	This study
BsPta_EcoRI_RBS_F	ATTTGGAATTCACTAGATAGTGGAGGTACTAGAATG CATCATCATCATCATCATGC	This study
BsPta_XhoI_PstI_R	TAACTCGAGCTGCAGTTACAAAGCCTGGGCAGC	This study
BsPta_Pho_R	/5Pho/TTACAAAGCCTGGGCAGC	This study

SmR_colony_F	GGC GAT GAG CGA AAT GTA GT	Roussou *et al.*, 2025
US_acs_colony_R	GGACAGTGGGGAAAAGATCA	Roussou *et al.*, 2025
DS_acs_colony_R	TTTCTTCATCCGTGGGACTC	Roussou *et al.*, 2025
US_ddh_colony_R	CTCACACTGGGGCAACTGTA	Roussou *et al.*, 2025
DS_ddh_colony_R	CGAACAGATAATCGGCATCA	Roussou *et al.*, 2025
SmR_integr_R	CAA CGC TAT GTT CTC TTG CTT T	Roussou *et al.*, 2025
UUS_acs_F	ACATCGGCTGGATGATGTTT	Roussou *et al.*, 2025
DDS_ acs_F	GGGCTTTGTTATTGGCTGAG	Roussou *et al.*, 2025
UUS_ddh_F	GCGACAGGAAAAGGAACAAA	Roussou *et al.*, 2025
DDS_ddh_F	GGAGCACAGCCTTGGATAAG	Roussou *et al.*, 2025
acs_integr_F	AGCAATCCGGAGAAGTTT	Roussou *et al.*, 2025
acs_integr_R	AATAAAGGCTCGGATGGC	Roussou *et al.*, 2025
ddh_integr_F	CTCAACCTTGATACCGCTATTTTAG	Roussou *et al.*, 2025
ddh_integr_R	ATGGGGACAGATTACTTGGTAAGTT	Roussou *et al.*, 2025
Term_backbone_F	TGCAGCCAGGCATCAAATAAAAC	This study

References:

Roussou, S., Pan, M., Krömer, J. O., & Lindblad, P. (2025). Exploring and increased acetate biosynthesis in Synechocystis PCC 6803 through insertion of a heterologous phosphoketolase and overexpressing phosphotransacetylase. *Metabolic Engineering*, *88*, 250–260. https://doi.org/10.1016/j.ymben.2025.01.008

Oliveira, P., & Lindblad, P. (2011). Novel Insights into the Regulation of LexA in the Cyanobacterium Synechocystis sp. Strain PCC 6803. *Journal of Bacteriology*, *193*(15), 3804–3814. https://doi.org/10.1128/JB.00289-11
